# Highly Filled Biocomposites Based on Metallocene Ethylene-Octene Copolymers with Wood Flour: Features of a Biodegradation Mechanism

**DOI:** 10.3390/polym17222970

**Published:** 2025-11-07

**Authors:** Anna K. Zykova, Anatoly A. Popov, Svetlana G. Karpova, Petr V. Pantyukhov

**Affiliations:** 1 Emanuel Institute of Biochemical Physics, Russian Academy of Sciences, Kosygina st. 4, Moscow 119334, Russia; 2 Higher Engineering School “New Materials and Technologies”, Plekhanov Russian University of Economics, Stremyanny per. 36, Moscow 117997, Russia

**Keywords:** biocomposites, ethylene-octene copolymer (EOC), wood flour, gel permeation chromatography (GPC), electronic paramagnetic resonance (EPR), biodegradation

## Abstract

This study examined the biodegradation process of highly filled biocomposites composed of ethylene-octene copolymer (EOC) and wood flour (WF) in varying proportions from 30 to 70 wt.%. The researchers analyzed the structure and characteristics of the samples before and after a 22-month soil-aging period. By employing techniques such as weight loss measurement, water absorption testing, optical microscopy, EPR spectroscopy with a radical probe, and gel permeation chromatography, the team identified fundamental patterns in oxidative and biological processes. The investigation revealed that the composition containing 40% WF exhibited the highest level of EOC degradation: polydispersity index increased from 2.7 to 4.5, and the Mw (weight-average molecular weight) decreased from 168 to 114 kDa. An explanation for this observation was proposed, suggesting that the phase structure significantly influences biodegradation, with the rate peaking when the interface area is maximized.

## 1. Introduction

Polyolefin thermoplastic elastomers are materials that combine polyolefin semi-crystalline thermoplastics and have amorphous elastomeric features with rubber-like and elastic properties; however, they can be mixed as melts in traditional thermoplastic equipment. There are several types of polyolefin thermoplastic elastomers: blends (thermoplastic polyolefins), dynamically vulcanized blends of ethylene-propylene random copolymer (EPM), ethylene propylene dienemonomer (EPDM) with an olefin (thermoplastic vulcanates), random block copolymers (ethylene-octene copolymer (EOC)), block copolymers (hydrogenate polybutadiene-isoprene-butadiene block copolymer), stereoblock polymers (stereoblock polypropylene), and graft copolymers (polyisobutylene-g-polystryrene) [1].

In this study, composites based on metallocene ethylene-1-octene copolymers (EOC) and wood flour (WF) were examined. The polymer used in this study was produced using a metallocene catalyst, which refers to a single-site or constrained-geometry catalyst. These catalysts contain a constrained transition metal sandwiched between one or more cyclopentadienyl ring structures to form sterically hindered polymerization sites [2].

The main differences between metallocene EOC and Ziegler–Natta EOC were considered in the literature [3]. Comparison analysis showed that metallocene and Ziegler-Natta copolymers with equal octene content (11 wt.%) had similar levels of short-chain branching (SCB), melt flow index, and density; however, the DSC assay revealed that the Ziegler copolymer melted at a higher temperature with an extra melting peak, which reflected the broader branch distribution in the heterogeneous Ziegler copolymer. This is highly correlated with a broader molecular weight distribution (MWD). In addition, the Ziegler copolymer had a higher degree of crystallinity, which was confirmed by the higher storage modulus and higher intensity of tg *δ_max_*. Other data emphasized the idea of the formation of “fringed micelles” in EOC, connected with the crystallization of polymer chains with a limited length of crystallized sequence [4]. According to these findings, these polymers had some crystallinity; nevertheless, the segments between branches were not long enough to be crystallized by the conventional chain-folding process, which resulted in a lower degree of crystallinity.

Recently, numerous scientific publications have focused on various aspects of EOC and related materials. These studies have explored a range of topics, including the characteristics of pure EOC with varying chain structures (blocky and random) [5] as well as the properties of pure EOC with different comonomer contents [6]. Additionally, researchers have investigated blends of polypropylene with EOC [7] and mixtures of low-density polyethylene and EOC [8]. The properties examined in these studies include mechanical [9] and thermal attributes [10], adhesion between EOC of different architectures (statistical and blocky) and polypropylene [11], and rheological behavior [12].

Limited research has been conducted on filled EOC composites, particularly regarding their biodegradation [13,14,15]. Previous studies have explored the biodegradability of composites made from EOC and natural fillers [16,17]. EOC was modified with maleic anhydride (MAH) and dicumyl peroxide [16]. The grafted EOC was subsequently combined with gelatinized waxy corn starch in various ratios. Specimens of the resulting materials were buried in natural soil. The results showed that a higher filler content in the EOC/starch composites led to an increased weight loss. The authors noted that pure EOC was not biodegradable in the soil. Scanning electron microscopy (SEM) revealed numerous large holes and cracks in the degraded surface morphology, especially in specimens buried for longer periods.

Another study examined the biodegradability of EOC/cornstarch composites using fungal cultures [17]. This research followed the ASTM G21 [18] standard to assess the resistance of synthetic polymeric materials to fungi. The fungal strains used in the microbiological tests included *Aspergillus niger* ATCC 9642, *Penicillium pinophilum* ATCC 11,797, *Chaetomium globosum* ATCC 6205, *Gliocladium virens* ATCC 9645, and *Aureobasium pullulans* ATCC 15,233. Substantial fungal growth was observed in the samples containing 30 wt.% and 40 wt.% starch. A higher starch content correlated with more significant fungal growth. As the starch levels increased, the film surface became rougher, displaying more and larger holes and cracks on the surface of the specimens due to starch consumption.

Many methods are available for assessing the biodegradability of polymer matrices in biocomposites. The first was the weight loss of the sample exposed to the soil media. Scientists are still using it [19,20,21], although this is not perfect because fungal growth inside samples leads to weight increase [22]. The second is the mold germination test [23], in which the Russian GOST 9.049-91 [24] standard is actively used to assess the resistance of materials to mold fungi [25]. A suspension of mold fungal spores (from the standard list) was sprayed onto the sample, and after 2 weeks, their germination on the surface was assessed. After four weeks, the size of the fungal colonies and the condition of the mycelium were assessed. The most commonly used method is to determine the volume of CO_2_ evaporated during the biodegradation of plastic materials in liquid soil media, the so-called Sturm method. It occurs according to ISO 14855-1:2012 [26] and ASTM D5988-18 [27], and it is used as the most reliable and standardized method for assessing biodegradability [28]. However, the most accurate reproduction of environmental conditions is the immersion of the samples in real soil. This method of aging the samples was chosen for this work.

Wood flour is a cheap, widely available industrial waste that is well known and described in the scientific literature. It mainly consists of cellulose (37–56%), lignin (18–22%), and hemicellulose (25–40%) [29,30]. In early works by Bledzki and Faruk, the high potential (strong mechanical properties) of biocomposites made of a polyolefin polymer matrix with WF was shown [31,32]. Recently, it was shown that by choosing an optimal grade of ethylene-vinyl acetate copolymer (EVA), it is possible to obtain a highly filled concentrate with WF (filled with 50–70 wt.%) [33]. It can be added to polyethylene as masterbatches by manufacturers of plastic goods without any technological difficulties. The same idea was used in this work, but instead of EVA, we used EOC. It also allows for the introduction of a large amount of vegetable filler into the polymer matrix. The effect of the filler type and content on the mechanical properties was published in a previous paper [15].

The aim of the current study was to establish a correlation between soil exposure and degradation of the polymer matrix. The correlation between the molecular weight distribution (MWD) of EOC and its exposure to soil (biodegradation) was established for the first time. We selected concentrations of biodegradable filler from 30 wt.% to 60 wt.% in biocomposites because at concentrations lower than 30 wt.%, the biocomposite is not biodegradable enough, while at concentrations higher than 60 wt.%, it has poor technological properties (MFI is very low).

## 2. Materials and Methods

Ethylene-octene copolymer (EOC), synthesized by LG Chem (Seoul, Republic of Korea) under the trademark POE Lucene LC670, was selected as the polymer matrix for highly filled biocomposites. The physicochemical properties were as follows: 1-octene content 38% mol., melt flow index 5.0 at 2.16 kg/190 °C, melting temperature 58 °C, density 0.870 ± 0.002 g/cm^3^, tensile strength 5.5 MPa, and elongation at break 900%.

Birch wood flour (WF) was selected as a filler for the biocomposites because of its high availability as a production waste in the wood-making industry. The chemical composition of this type of filler has been described previously [34]. In brief, WF contained cellulose (37–56%), lignin (18–22%), hemicellulose (25–40%), and trace amounts of proteins, fats, and ash. The filler was ground and sieved by a testing sifter Matest A059-02KIT (Matest, Arcore, Italy). The mesh holes of the laboratory sieves were 500, 250, and 200 μm. The smallest fraction (0–200 μm) was chosen for the preparation of biocomposites.

The filler contents of the prepared biocomposites were 30, 40, 50, and 60 wt.%. Biocomposites were obtained using heated mixing rolls Dongguan BaoPin UBL-6175BL (Dongguan BaoPin International Precision Instruments Co., Ltd., Dongguan, China)at 160 °C for 5 min. The filling pieces were crushed using a rotary knife mill Vibrotechnik RM-120 (Vibrotechnik, Saint Petersburg, Russia) and then pressed using a manual hydraulic press VNIR PRG-1–10 (VNIR, Moscow, Russia) at 135 °C under a load of 7 kN for 3 min, followed by quick cooling. The obtained specimens were round, with a diameter of 7 cm. The thickness of the films varied from 100 μm for composites with 30 wt.% filler to 1000 μm for composites with 60 wt.% filler. Thin films are better for studying the dispersion of particles, but the high-melt viscosity of highly filled biocomposites did not allow the formation of thin films. In all experiments, standardization was performed according to the thickness of the samples.

Biodegradation tests were carried out under natural conditions in accordance with ASTM D 5988-12 [35]. The soil mixture consisted of equal amounts of garden soil, horse manure, and sand. The soil humidity was maintained at 60 ± 5%. The soil was prepared according to the method previously applied in [36]. The samples were placed vertically in the mixed soil and held for 22 months. After removal from the soil, the samples were washed in cold water and air-dried at 25 °C for 2 weeks. The differences in mass, appearance, water absorption, and molecular characteristics of the initial and exposed biocomposites were analyzed.

To detect the changes in morphology and establish the microbiological deterioration of the exposed specimens, optical microscopy in reflected light mode was applied. An optical microscope Carl Zeiss Axio Imager Z2M (Zeiss, Oberkochen, Germany) with the image software AxioVision ver. 4.7.1 at magnification 200× was employed.

The stability of the initial composites in aqueous media was investigated according to the ISO 62:2008 standard [37]. Three film specimens of each composition were placed in test tubes containing distilled water and aged at 30 °C. The experiment was extended to 31 days until full water retention.

The quantity of leachable water-soluble substances in the fillers was measured by the difference between the mass of the initial samples and the mass of the samples dried to a constant mass (at 80 °C) at the end of the experiment (Δm = m − m_0_). The weight loss curves of the composites after the water absorption test were normalized by the measured quantity of leachable water-soluble substances.

To characterize the amorphous regions and segmental mobility of the polymer chains of the selected initial and exposed samples, electronic paramagnetic resonance (EPR) spectra were recorded using an EPR-V automated spectrometer FRCCP RAS (Moscow, Russia). To avoid saturation effects, the microwave power in the resonator cavity did not exceed 7 mW. The stable nitroxyl radical TEMPO [(2,2,6,6-Tetramethylpiperidin-1-yl)oxyl] was used as the probe. The radicals were introduced into the samples from the gas phase at a temperature of 60 °C. The values of the correlation time for probe rotation were obtained from the EPR spectra.

To determine the effect of soil on the macromolecular characteristics, gel permeation chromatography was performed. In the first stage, the extraction of EOC from the initial and exposed specimens was conducted in 1,2,4-trichlorobenzene (TCB) based on a modified approach of [38,39]. The EOC solutions extracted from the initial and exposed composites were separated from the hard particles by sedimentation and then by a paper filter. The liquid EOC solutions were analyzed by high-temperature gel permeation chromatography (HT-GPC). Analysis was performed on a Waters GPCV 2000 system (Waters, Milford, MA, USA), and separation was performed using a Styragel HT 6 E column (diameter to length: 7.8 × 300 mm) in series at 140 °C and a TCB flow rate of 1 mL/min. The components were detected using a refractive detector. The GPC system was calibrated using polystyrene standards with known M_w_ and M_n_.

The work was carried out using scientific equipment from the Center of Shared Usage «New Materials and Technologies» of the Emanuel Institute of Biochemical Physics, Russian Academy of Sciences, and the Joint Research Center of Plekhanov Russian University of Economics. The molecular characteristics of the initial and exposed composites were determined at the Petrov Institute of Plastics.

## 3. Results

**Figure 1:**
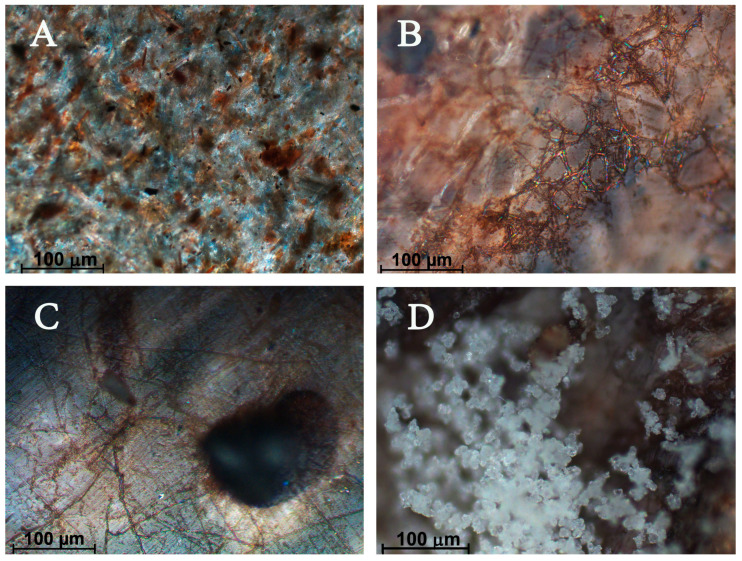
Photomicrographs of the film samples with 30% of WF. (**A**)—initial soil exposure; (**B**–**D**)—after 22 months of soil exposure.

**Figure 2:**
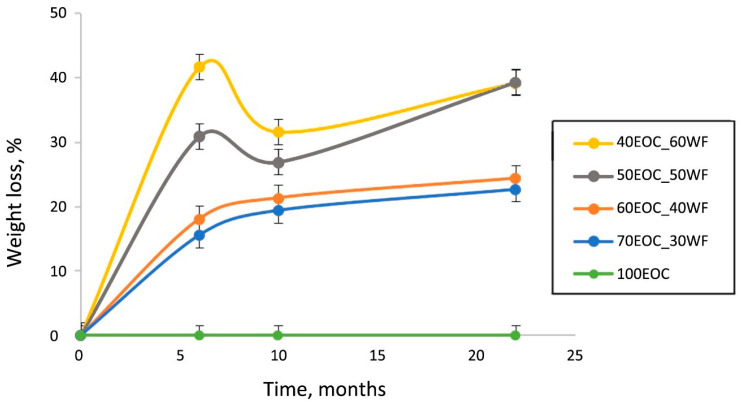
Weight loss dynamics of biocomposites during exposure in soil.

Biodegradation studies under open soil conditions are presented in Figure 1 and Figure 2. Photomicrographs demonstrate the biodegraded parts of biocomposites (Figure 1B–D) in comparison with the initial sample (Figure 1A). We found surface mycelium (Figure 1B), torn out filler particles (Figure 1C), and aerial mycelium (Figure 1D).

Figure 2 shows that, as the filler content increased, the weight loss of the composites increased. Composites with the highest filler contents (50 and 60 wt.%) exhibited a significant increase in weight loss of up to 30 and 41%, respectively, during the first period of the soil test. This effect may be attributed to the washing out of water-soluble substances and the degradation of the filler under the action of enzymes. Later, the change in weight of these specimens became more stable owing to the accumulation of microorganisms (filling of the voids that emerged after the filler was washed out). The composites with small amounts of filler (30 and 40%) demonstrated a slight weight loss dynamic without a significant initial increase, which was observed in composites with higher filler content. Pure EOC specimens showed no evident weight loss during 22 months, which is consistent with previous studies [16].

**Figure 3:**
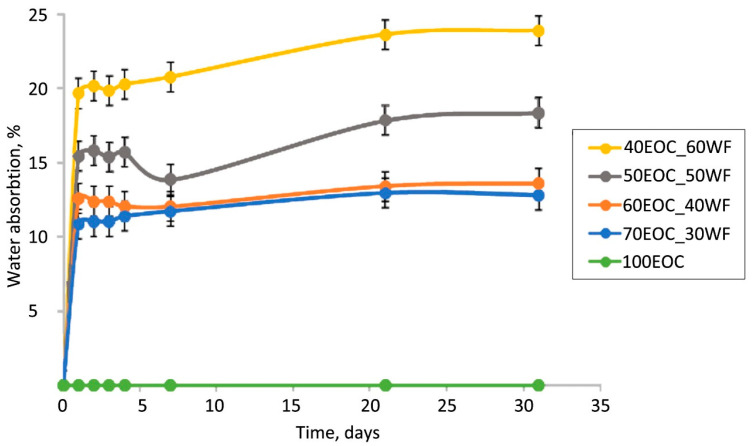
Kinetic curves of water absorption of biocomposites.

Similar conclusions can be drawn from the water absorption capacity test of the initial composites (Figure 3). The distribution of the kinetic curves of the EOC composites with varied filler contents is in agreement with the results of the biodegradation tests. The addition of lignocellulosic filler caused an increase in the water absorption of the composites from 0% to 20% at the highest filler content. It is known that WF particles contain fine pores and hydrogen bonding sites [40], which are able to absorb water. Water absorption is an integral process that includes the absorption of water and the desorption of water-soluble components in WF. On the first day, the samples absorb an amount of water proportional to their WF content. After the first day, the kinetic curves were observed to reach a plateau, indicating that the active stage of water absorption finished. After the first stage, a small decrease in water absorption was observed for biocomposites with 40 wt.% and 50 wt.% of WF. Obviously, it was caused by the desorption of water-soluble components into the water. The process of desorption occurs in all biocomposites, but it is compensated by water absorption; that is why it cannot be seen in kinetic curves with 30 wt.% and 60 wt.% of WF. After 7 days, water absorption slightly increased, and this increase was more pronounced for biocomposites with 50 wt.% and 60 wt.% of WF, where the WF formed its own continuous phase.

The similarity of the dependences shown in Figure 2 and Figure 3 provides information regarding the distribution of filler particles in the volume of the polymer matrix. The proximity of the kinetic curves with 30% and 40% filler indicated the encapsulation of WF particles inside the matrix. An increase in the filler content above 40% leads to particle aggregation and the formation of its own continuous phase. A further increase in the filler content up to 60% creates an opportunity for greater particle aggregation. This leads to a decrease in the filler particle content in the volume of the polymer phase. There were two independent phases: a hydrophobic polymer phase and a hydrophilic filler phase. An interpenetrating network structure is formed.

The phase structure of a composite, made of incompatible components, is well known and has been widely discussed, with J.R. Millar introducing the term “Interpenetrating polymer networks” in 1960 [41,42]. The only difference between the obtained data and those reported in the literature is the larger filler content (over 40 wt.%). This is the amount necessary for the formation of a continuous phase of the second component. Most often, interpenetrating networks are formed when the content of the second component reaches 30 wt.%. Composites based on polyethylene with polypropylene and their blends with natural fillers, including WF, have a similar structure [22,43,44]. This difference can be explained by the structure of the EOC. It has a low content of defect crystallites (approximately 6%) and a melting point of ~50 °C. The crystalline phase did not have a significant impact on EOC properties. This is the difference between EOC and semicrystalline polyolefins, which contain a significant proportion of crystallites. Polyolefins are compounded with natural fillers at temperatures above the melting point of the polymer. Upon further cooling, the formed crystallites displaced the filler into an amorphous phase. In this case, the quantity of filler in the amorphous phase is higher, and the formation of a continuous filler phase begins at filler concentrations higher than 40%.

The continuous phase of the WF at 50–60 wt.% concentration allowed the water and water-soluble substances to penetrate into the bulk of the composite. For that reason, the highest weight loss (Figure 2) was found for these composites (50EOC-50WF and 40EOC-60WF). However, weight loss is not an ideal parameter for evaluating biodegradability. This is an integral value that takes into account both the loss of the filler due to the leaching of water-soluble components and consumption by the microbiota and biofouling from the surface and in the volume of the sample, leading to an increase in the mass of the sample [22].

Visual observation of the samples after 10 months showed that as the filler content increased, the biodeterioration of the composites increased. At small amounts of filler content, the colonization of microorganisms was not evident, and the area of the surface filled by hyphae was not significant. It was noted that fungal growth depended on both the filler content and its acceptability to the enzymes of the microorganisms. Fungal mycelium was mainly concentrated in discontinuities between the wood particles and polymer matrix near the specimen surface. It was concluded that, as the filler content increased (more than 50 wt.%), the surface became more defective. In addition, increased WF content resulted in higher weight loss and biodegradation, which caused the formation of cracks and holes, exposing the composite to microorganisms [45].

Notably, the mechanical properties of the obtained biocomposites in a prior study also showed a gap between 40 and 50% of WF [15]. Modulus of elasticity increased from 50 MPa for 60EOC-40WF to 100 MPa for 50EOC-50WF. This increase in elasticity was explained by structure transformation in biocomposites from a dispersed system to an interpenetrating network in that area, but there was no evident proof for that hypothesis obtained by other research methods. In this work, our results confirmed the hypothesis by weight loss and water absorption.

**Figure 4:**
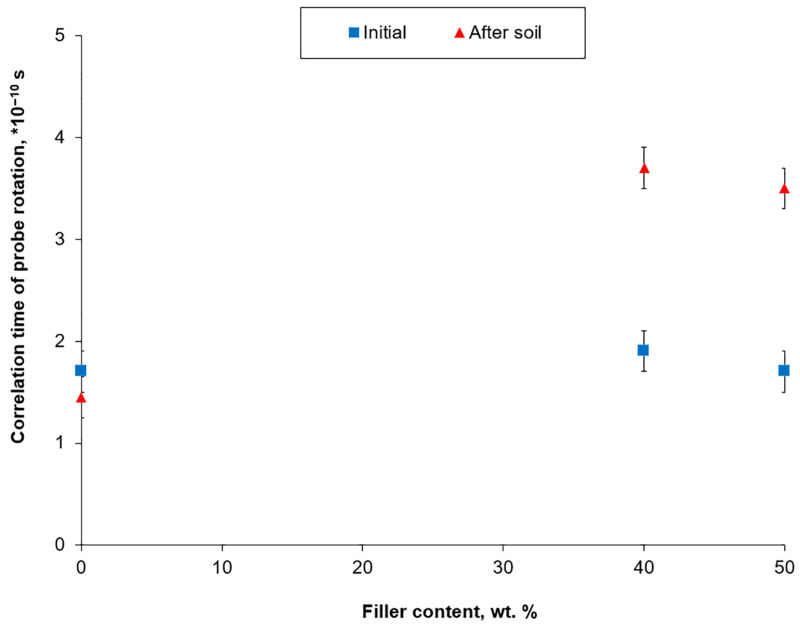
The correlation time of probe rotation in initial and exposed samples in dependence on filler content.

The analysis of EPR spectra of the initial and several exposed samples in terms of the correlation time of probe rotation is presented in Figure 4. The same values of the probe correlation time (1.8 ± 0.2) × 10^−10^ sec for the initial samples (for the pure copolymer and for biocomposites containing 40% and 50% WF) indicate the following: first, the probe is in the amorphous phase of the polymer and, second, that the presence of the filler does not affect the segmental mobility of the EOC macrochains. After exposure to the soil, the correlation time of the probe for the pure copolymer did not change and was within the experimental error. This indicated that no changes occurred in the amorphous phase of the unfilled sample. A different picture is observed for the samples with WF. The impact of environmental factors, such as oxygen and microbiota enzymes, on the entire volume of filled samples is accompanied by oxidative destruction. Oxidation of the copolymer macrochains occurs in the amorphous phase, where oxygen-containing compounds accumulate. They increase the polarity of the polymer matrix, which is accompanied by a slowdown in segmental mobility. This explains the increase in the correlation time of the stable radical probe. This effect is well known for the oxidation of various polyolefins under autooxidation, ozonation, and UV aging conditions.

**Figure 5:**
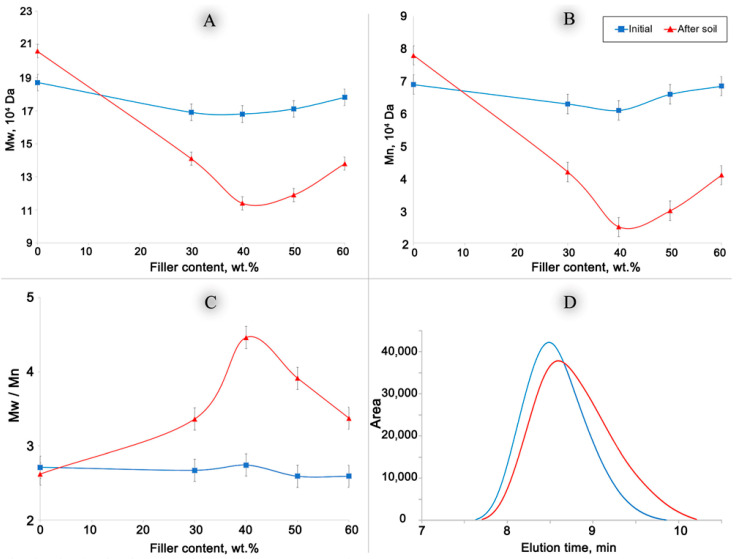
The changes in molecular parameters (M_w_, M_n_, PDI) of EOC polymer matrix in dependence on filler content before and after biodegradation test ((**A**), (**B**), (**C**), respectively) and typical raw-data chromatograph curves for initial and exposed samples (**D**).

The polymer matrix was investigated using the HT-GPC method to determine the effect of soil exposure on the molecular characteristics of the EOC. The curves of the molecular characteristics of the initial and exposed composites with different filler contents are shown in Figure 5. M_w_ is the weight-average molecular weight, M_n_ is the number-average molecular weight, and PDI is the polydispersity index.

Alterations in the molecular parameters of the polymer matrix (EOC) of specimens with varying filler content before and after exposure to soil for a duration of 22 months were considered (Figure 5). With the addition of WF, the molecular parameters of the EOC did not change significantly. The slight reduction in M_w_ and M_n_ values may be caused by the mechanochemical destruction of macromolecules at the processing stage. The molecular parameters of synthetic polymers without fillers were stable. The slight increase in M_w_ and M_n_ values after soil exposure can potentially be considered negligible. In all the biocomposites with WF, the molecular mass of the EOC matrices was significantly reduced. The greatest reduction was observed for the biocomposite containing 40% WF. The initial value Mw = 16.8 × 10^4^ decreased to 11.4 × 10^4^, and Mn value decreased from 6.1 × 10^4^ to 2.5 × 10^4^. The polydispersity index of this biocomposite exhibited the highest value following exposure to soil, increasing from 2.7 to 4.5. The shift in the MWD of the sample with 40% WF to lower molecular weight values with higher polydispersity after exposure to soil is shown in Figure 5D.

The obtained data suggest that after exposure to soil, the polymer matrix of biocomposites undergoes significant biodegradation, and the rupture of macromolecules, reduction in molecular weight, and increase in polydispersity are obvious evidence of polymer degradation. This result was the main conclusion of this study.

Additional experimental observations warrant further explanation. The addition of 30% and 40% of the filler resulted in a significant decrease in M_w_ and M_n_ and an increase in polydispersity. However, at higher filler concentrations (50% and 60%), the nature of the dependence changed with increasing M_w_ and decreasing PDI. This phenomenon can be attributed to the interface. At lower concentrations (30–40%), the filler was uniformly distributed throughout the polymer matrix, forming a dispersed phase. The interfacial area between the filler and the polymer reached its maximum at 40%. A further increase in the filler concentration leads to the formation of agglomerates and the initiation of its own phase, resulting in a decrease in the interfacial area. Previous research has demonstrated that the interfacial area in a biocomposite accelerates chemical reactions (including destructive processes such as oxidation, ozonolysis, biodegradation, etc.) in the polymer matrix [22]. This phenomenon is attributed to the nonequilibrium state of the surface layer and the presence of overstressed bonds on the polymer surface. This mechanism may elucidate the dependencies observed in Figure 5.

## 4. Conclusions

The main objective of obtaining composites based on degradation-resistant synthetic polymers with natural fillers is to create favorable conditions for their decomposition after the end of the product’s service life. The presence of a filler allows the main destructors (oxygen, water, microorganisms, and their metabolites) to penetrate the volume of the synthetic polymer. Many properties of composites are determined by the amount of the filler introduced. Thus, an increase in the content of WF is accompanied by an increase in the water absorption of samples, greater weight loss after exposure to soil, and an increase in colonization by microorganisms. These characteristics allowed us to judge the ability of the material to biodegrade. However, quantitative criteria primarily characterize the decomposition of the filler. Therefore, it is worth paying closer attention to EPR spectroscopy and HT-GPC data, which characterize the changes occurring in the synthetic polymer. Both methods showed no significant changes in the pure copolymer without filler after aging in the soil for 22 months. However, in WF-based biocomposites, destructive oxidative processes accompanied by the rupture of copolymer macromolecules were detected. The greatest changes in EOC were observed in the biocomposites with WF contents of 40 wt.%: Mw (weight-average molecular weight) decreased from 168 to 114 kDa, and Mn (number-average molecular weight) decreased from 61 to 25 kDa; polydispersity index increased from 2.7 to 4.5. At this WF content in the biocomposite, the polymer is a dispersion medium and the filler is a dispersed phase. The largest phase separation area was observed for the 40 wt.% of the filler. At a higher filler content, WF forms an extended phase, and a structure of interpenetrating networks is formed. The contact area between the phases decreased, which was accompanied by a slowdown in the destructive process.

Thus, the phase structure of the biocomposite plays a significant role in biodegradation kinetics because it defines the phase separation area. Increasing the phase-separation area makes it easier for destructors to penetrate into the volume of the synthetic polymer. In addition, it should be noted that polymer macromolecules located on the surface have increased reactivity [22], which creates an additional opportunity for destructive processes to occur.

## Data Availability

The original contributions presented in this study are included in the article. Further inquiries can be directed to the corresponding author.

## Abbreviations

The following abbreviations are used in this manuscript:

EOC	Ethylene-Octene Copolymer
WF	Wood Flour
EPR	Electronic Paramagnetic Resonance
GPC	Gel Permeation Chromatography
HT-GPC	High Temperature Gel Permeation Chromatography
PDI	Polydispersity Index
